# Development and validation of a real-time risk prediction model for acute kidney injury in hospitalized pediatric patients

**DOI:** 10.1007/s12519-025-00950-2

**Published:** 2025-07-30

**Authors:** Chao Zhang, Chen Wang, Qin-Shi Hu, Xi-Ming Xu, Ruo-Hua Yan, Xiao-Lu Nie, Ya-Guang Peng, Hai-Ping Yang, Yao Song, Xue-Jun Yang, Xiao-Xia Peng

**Affiliations:** 1 Department of Clinical Epidemiology and Evidence-Based Medicine, National Center for Children Health, Beijing Children’s Hospital, Capital Medical University, No.56 Nanlishi Road, Xicheng District, Beijing, 100045 China; 2 Big Data Center for Children’s Medical Care, Children’s Hospital of Chongqing Medical University, Chongqing, China; 3 Department of Nephrology, National Clinical Research Center for Child Health and Disorders, Ministry of Education Key Laboratory of Child Development and Disorders, Chongqing Key Laboratory of Pediatric Metabolism and Inflammatory Disease, Children’s Hospital of Chongqing Medical University, No. 136 Zhongshan Er Road, Yuzhong District, Chongqing, 400014 China; 4 Department of Biostatistics, University of Michigan, Ann Arbor, USA

**Keywords:** Acute kidney injury, Machine learning, Pediatric, Predicting, Prevention, Real-time prediction, Risk

## Abstract

**Background:**

Acute kidney injury is associated with a prolonged hospital stay and high mortality for pediatric patients. The previous prediction models are based on a pre-defined time window which may affect its feasibility in clinical practice. This study aimed to develop and validate a real-time acute kidney injury risk prediction model for hospitalized pediatric patients.

**Methods:**

Based on a retrospective cohort composed of eligible pediatric patients hospitalized in Beijing Children’s Hospital and Chongqing Children’s Hospital, a machine learning model to predict acute kidney injury occurrence was developed and validated. The prediction model was established using a stacking technique to combine three base learners including XGBoost, LightGBM, and CatBoost. Particle swarm optimization algorithm was used to tune hyperparameters. Next, we assessed the performance of the prediction model using the area under the receiver-operating curve and the area under the precision–recall curve.

**Results:**

A total of 26,671 patients were included (20,967 in the derivation set, 5242 in the internal validation set, and 462 in the external validation set) contributing to 36,828 hospitalizations. The new proposed model had excellent performance for predicting any acute kidney injury within 24 hours. Both the internal [area under the receiver-operating curve (AUROC) was 0.851, area under the precision–recall curve (AUPR) was 0.322] and external validation set (AUROC was 0.869, AUPR was 0.270) approved the model’s feasibility in predicting the risk of acute kidney injury.

**Conclusion:**

The established risk predicting model can be used for real-time prevention of acute kidney injury in hospitalized pediatric patients.

**Graphical abstract:**

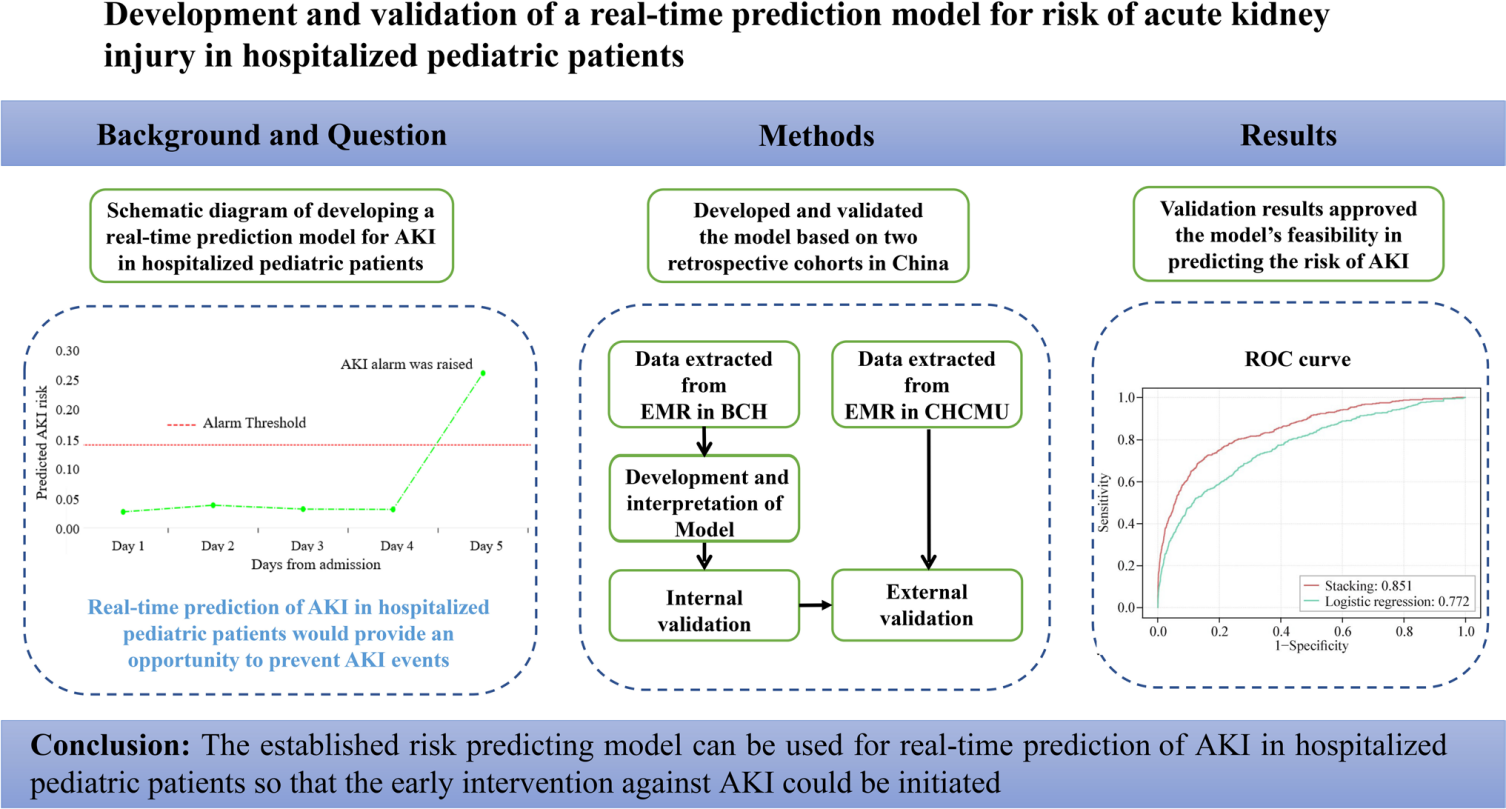

**Supplementary Information:**

The online version contains supplementary material available at 10.1007/s12519-025-00950-2.

## Introduction

Acute kidney injury (AKI) is an adverse event characterized by a rapid decrease in renal excretory function that has been reported to be associated with a prolonged hospital stay and mortality [1, 2]. According to the results of existing adult study [3], theoretically, early prediction of AKI in hospitalized pediatric patients would provide an opportunity to prevent AKI events and reduce adverse outcomes.

For this reason, the development of AKI prediction models for pediatric inpatients has recently been of concern. There are efforts to predict inpatients’ AKI from electronic medical records (EMR) data using various machine learning and deep learning techniques via two common ways [4]. One approach is to predict the risk of AKI within 24 hours, 48 hours or longer after a fixed time point based on clinical data [5–11]. Another is based on clinical data from 48 hours or other prediction windows before onset [12]. However, the prediction models based on a pre-defined time window rather than real-time clinical changes might affect the feasibility of transforming these AKI models into clinical practice [13].

Accordingly, the 15th Acute Dialysis Quality Initiative (ADQI) consensus conference has identified AKI as a target for risk prediction and recommended the development of AKI alert systems that will provide predictive AKI risk up until the occurrence [13]. The primary goal of this study is to develop a real-time AKI risk prediction model for pediatric patients using a machine learning model.

## Methods

### Study design and participants

The present study was performed in accordance with the recommendations laid out in the World Medical Association Declaration of Helsinki. Ethics approval was obtained from the Institutional Review Boards of the Beijing Children’s Hospital (IRB No. [2022]-E-207-Y) and the Institutional Review Boards of the Children’s Hospital of Chongqing Medical University (IRB No. 2023–488). Written consent was waived by the IRB as only retrospective data was used.

This study was performed using two retrospective cohorts from two pediatric hospitals in China. The Beijing cohort consisted of all patients hospitalized at Beijing Children’s Hospital, Capital Medical University from 2015 to 2020, with which we trained and performed an internal validation of the AKI prediction model. The Chongqing cohort consisted of all patients hospitalized at Children’s Hospital of Chongqing Medical University from Jan to June 2023, with which we performed an external validation of the AKI prediction model. Both Beijing Children’s Hospital and Children’s Hospital of Chongqing Medical University are tertiary care university teaching hospitals, located in North China and Southwest China respectively and are using different EMR systems. The same exclusion criteria were followed in both hospitals: (1) patients without at least two creatinine tests; (2) age under 28 days; (3) estimated glomerular filtration rate (eGFR) < 15 mL/minute/1.73 m^2^ or baseline creatinine > 4 × upper limit of normal (ULN) or diagnosed with chronic kidney disease (CKD) at admission, and (4) AKI developed within 24 hours of admission.

This manuscript is written following the Transparent Reporting of a Multivariable Prediction Model for Individual Prognosis or Diagnosis (TRIPOD) checklist [14].

### Data abstracting and preprocessing

First, a list of potential variables involved in model training was designed based on a review of prediction models of pediatric AKI and expertise in AKI occurrence and progress. Next, these variables were abstracted from EMR. The variables included demographics, comorbidities at admission, hospital procedures, drugs, laboratory testing data and vital signs. The variables with missing values exceeding 80% are excluded from statistical analysis. An advantage of the ensemble learning model is that redundant variables can be ignored, so a total of 75 variables were included in the model training to obtain a better predictive performance. A summary of these variables can be found in Supplementary Table 1. However, considering the ease of implementation of the model, we also performed feature selection using the genetic algorithm to obtain a simplified model. The results of the feature selection and the predictive performance of the simplified model are shown in Supplementary Table 2 and Supplementary Fig. 1.

Each predictor was classified as either a static or dynamic variable [15]. The static variables were defined as time-invariant during hospitalization and included demographics, comorbidities at admission and surgical procedures. Dynamic variables were values that were updated on a daily basis and included vital signs, laboratory testing values and drugs [16, 17]. All data in the observation window were used to predict AKI risk by merging dynamic variables in 24 hours with static variables.

The missing variables at baseline were imputed as median value [18, 19]. For missing values after admission, imputation was performed by the “last observation carried forward” method [20].

Numeric variables were scaled as follows:

$${\text{X}}_{scaled}=\frac{\text{X }-\upmu (\text{X})}{\upsigma (\text{X})}$$

Where μ(X) denotes the mean and σ(X) denotes the standard error of the feature X [21, 22].

**Fig. 1:**
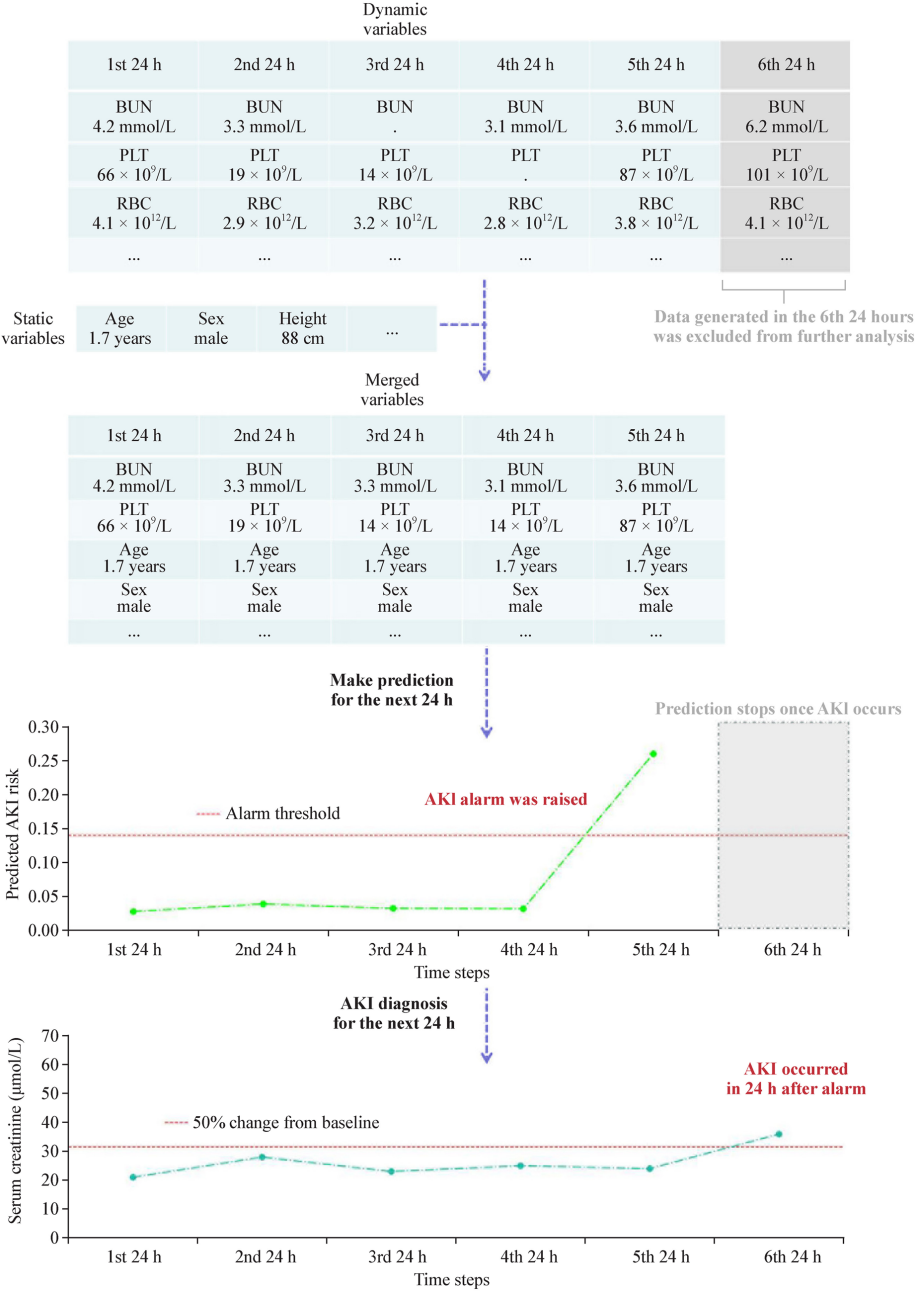
Diagram illustrating data preprocessing and the prediction window. A child developed AKI in the 6th day after admission was used as an example. A risk above 0.14 (corresponding to 50% precision) was the threshold above which AKI was predicted. *AKI* acute kidney injury, *BUN* blood urea nitrogen, *PLT* platelet, *RBC* red blood cell

The flow of data preprocessing as well as making real-time predictions is shown in Fig. 1. First, the updated dynamic variables for each patient are combined with the static variables to make a set of predictor variables (Fig. 1a). This way every patient in the data set was represented by a sequence of time steps (Fig. 1b) [23]. For each time step, the model would predict the occurrence of AKI in the next 24 hours (Fig. 1c). As a real-time prediction model, it will keep predicting AKI risk until actual diagnosis of AKI or discharge. Using the true test value of serum creatinine, we can determine whether the model is making correct predictions (Fig. 1d).

### Definition and classification of acute kidney injury

AKI status was labeled according to the Kidney Disease Improving Global Outcomes (KDIGO) criterion [24]. Due to the relative sparsity of urine output data on the general hospital wards, urine output criteria for AKI were not considered. According to KDIGO guidelines an increase in serum creatinine of 0.3 mg/dL (26.5 μmol/L) within 48 hours or an increase in serum creatinine of 1.5 × the baseline creatinine level was identified as AKI. The same enzymatic method of serum creatinine measurement ensures diagnostic consistency between the two hospitals. Baseline creatinine was determined as the first creatinine measurement on admission. Onset time of AKI was labeled as the time of creatinine measurement on AKI diagnosis. The full-age-spectrum (FAS) equation was used to estimate glomerular filtration rate [25]. Two prediction windows were considered in this study, i.e., AKI within 24 hours or AKI within 24–48 hours.

### Model training

Considering that the model is designed to predict the risk of AKI in real-time, it is evaluated daily for each patient during hospitalization. There are only 3.0% of time steps associated with a positive AKI label, indicating that the AKI prediction task is class-imbalanced.

To address class-imbalance, the technique of ensemble learning was employed [26, 27]. Ensemble learning combines several base models to improve learning efficiency. It has been shown that boosting models achieved good performance in AKI prediction models [28–31]. Thus, XGBoost, LightGBM and CatBoost were selected as base learners and were then combined using a stacking technique. Meanwhile, a logistic regression model was built for comparison with the stacking model.

To explore the best hyper-parameters of the stacking model, the particle swarm optimization (PSO) algorithm was used. This is an evolutionary algorithm designed by simulating the predatory behavior of a flock of birds [32]. Each base learner needs to be fine-tuned because they all have several hyperparameters. The PSO algorithm is expected to optimize the model more efficiently [33, 34]. An overview of the workflow for the modeling procedure is shown in Supplementary Fig. 2. The hyperparameter values are shown in Supplementary Table 3. The stacking model was fine-tuned within the derivation set through cross-validation to avoid overfitting.

### Model validating

The Beijing cohort was split into derivation and internal validation sets in such a way that information from a given patient was present only in one split. The derivation set was used to develop the proposed models and to tune the hyperparameters based on cross-validation methodology. To ensure independence between the derivation and internal validation set, we sampled based on patients instead of admission cases. In particular for patients with multiple admissions, all of their records were partitioned together either in the training or the test set.

The main performance indices used were: sensitivity (also known as recall), specificity, positive predictive value (PPV, also known as precision), negative predictive value (NPV), F1-score, the area under the receiver-operating curve (AUROC) and the area under the precision–recall curve (AUPR). Considering the feasibility of clinical prediction models in clinical practice, a cut-off value was chosen for the model to achieve at least 0.1 precision so that the number of false positives should ultimately not be a significant barrier to further implementation [23, 35]. A 10 × tenfold cross-validation was also conducted to obtain stable estimations of performance indices [36, 37].

### Model explainability

The ensemble learning model cannot directly offer any explanations regarding the clinical meaning of features. We utilized the SHapley Additive exPlanation (SHAP) value, derived from game theory to determine the importance of features in our prediction model [38]. This is calculated by taking the average marginal contribution of all possible coalitions for a feature value. At the same time, the SHAP plots are figured to show the individual-level SHAP value and the importance of each variable in the prediction model.

### Statistical analysis

Data preprocessing was conducted using SAS (version 9.4) and R (version 4.1.0). Descriptive statistical analyses were performed using SAS (version 9.4). The machine learning models were built and assessed using Python (version 3.10.4). Specifically, the categorical variables were summarized as counts and percentages (%), whereas real-time variables were described using median values and interquartile ranges (IQR). The differences between groups of categorical variables were compared using Chi-square or Fisher’s exact tests, and that of real-time variables were compared with the *t*-test or Wilcoxon rank sum test.

## Results

### Baseline participant characteristics

**Fig. 2:**
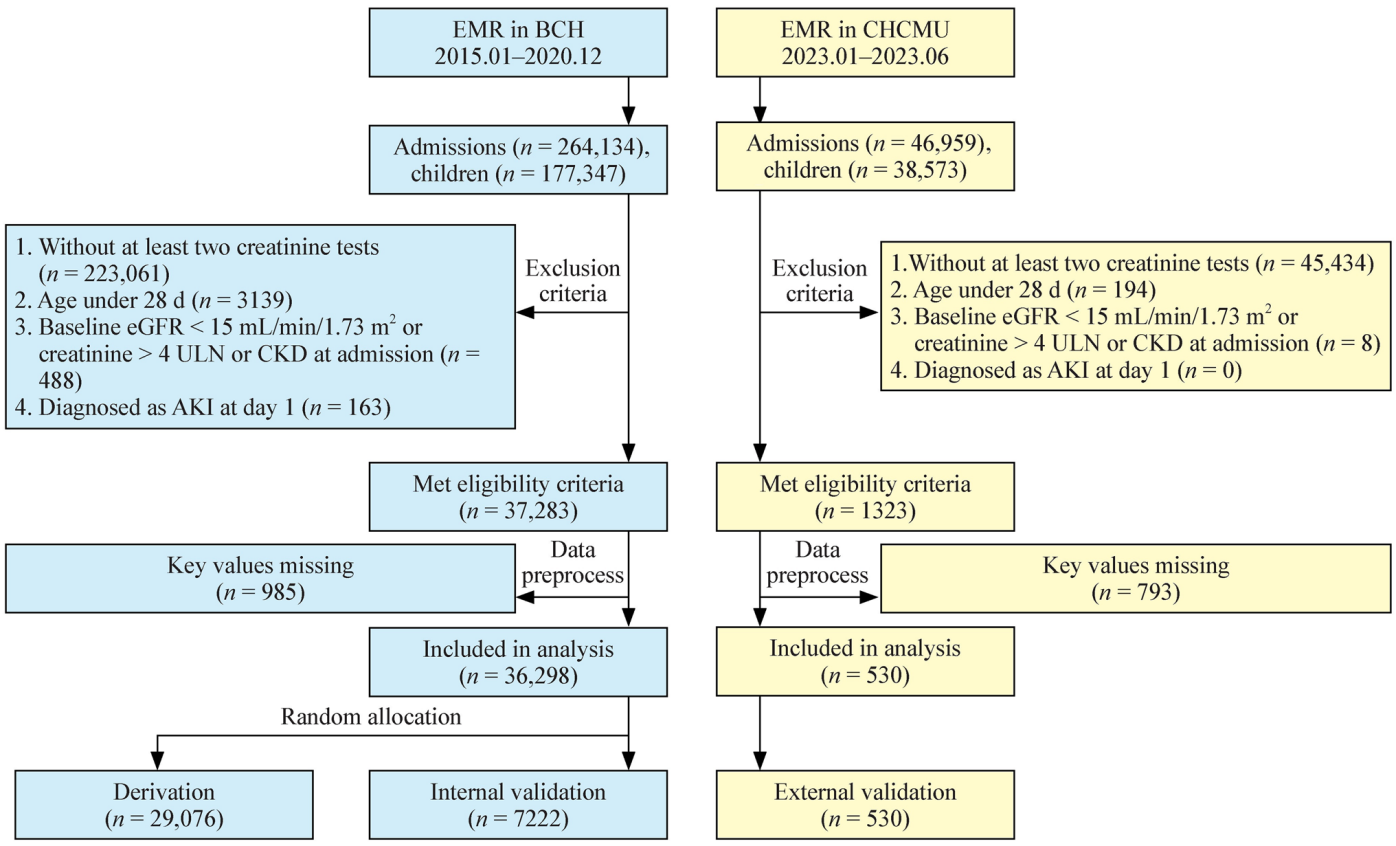
Study design flow chart. *AKI* acute kidney injury, *BCH* Beijing Children’s Hospital, *CHCMU* Children’s Hospital of Chongqing Medical University, *CKD* chronic kidney disease, *eGFR* estimated glomerular filtration rate, *EMR* electronic medical records, *ULN* upper limit of normal

According to participant selection criteria, 26,209 pediatric patients were selected from the Beijing cohort of 177,347 inpatients, resulting in 36,298 hospitalizations. Among 36,298 hospitalizations, 29,076 (80%) were randomly assigned to a derivation set from which a predictive model for pediatric AKI risk would be derived. The remaining 20% of hospitalizations comprised a validation set for model validation. It should be noted that randomization was performed at the patient level so that no patient overlapped between the derivation and internal validation set. In addition, 462 inpatients (resulting in 530 hospitalizations) were filtered from a total of 46,959 in the Chongqing cohort and were used as an external validation set for the prediction model. A participant flow diagram is shown in Fig. 2.

As shown in Table 1, 57.7% of 29,076 hospitalizations in the derivation set were boys with a median age of 4.7 (IQR: 1.8–9) years. Median hospital stay was 13 (IQR: 8–18) days. The pediatric patients with comorbidities of hypertension and diabetes accounted for 6.7% and 4.9%, respectively. In addition, pediatric patients exposed to non-steroidal anti-inflammatory drugs or anti-tumor agents accounted for 32.9% and 22.0%, respectively. In the external validation set from the Chongqing cohort, similar demographic characteristics were observed. However, there are also distinct clinical characteristics compared to the training set, including shorter durations of hospital stay (median: 8, IQR: 6–13), different spectrum of comorbidities upon admission and varied drug exposures.

**Table 1:** The demographic and clinical characteristics of participants

Variables	Training set based on Beijing cohort	Internal validation set based on Beijing cohort	External validation set based on Chongqing cohort
Number of children	20,967	5242	462
Number of hospitalizations	29,076	7222	530
Gender (male), *n* (%)	16,780 (57.7)	4270 (59.1)	307 (57.9)
Age (y), median (IQR)	4.7 (1.8, 9.0)	4.8 (1.9, 8.7)	5.9 (2.8, 10.2)
Hospital stay (d), median (IQR)	13 (8, 18)	13 (8, 18)	8 (6, 13)
Baseline Cr (μmol/L), median (IQR)	27 (21, 37)	26 (21, 36)	32 (25, 40)
Baseline eGFR (mL/min/1.73 m^2^), median (IQR)	114 (85, 143)	114 (82, 143)	117 (97, 136)
Comorbidities, *n* (%)			
Diabetes	1414 (4.9)	339 (4.7)	1 (0.2)
Heart failure	1145 (3.9)	278 (3.9)	4 (0.8)
Hypertension	1959 (6.7)	523 (7.2)	6 (1.1)
Pulmonary hypertension	497 (1.7)	149 (2.1)	26 (4.9)
Respiratory failure	1904 (6.6)	482 (6.7)	36 (6.8)
Septicemia	1216 (4.2)	281 (3.9)	90 (17.0)
Drugs, *n* (%)			
ACEI	1593 (5.5)	412 (5.7)	25 (4.7)
Acyclovir	1623 (5.6)	407 (5.6)	3 (0.6)
AGs	553 (1.9)	117 (1.6)	21 (4.0)
Anti-tumor	6392 (22.0)	1587 (22.0)	242 (42.1)
ARB	55 (0.2)	19 (0.3)	13 (0.9)
Diuretics	1162 (4.0)	285 (4.0)	117 (20.2)
NSAID	9562 (32.9)	2396 (33.2)	2 (15.7)

*IQR* interquartile range, *ACEI* angiotensin-converting enzyme inhibitor, *AGs* amino glycosides, *ARB* angiotensin receptor blocker, *Cr* creatinine, *eGFR* estimated glomerular filtration rate, *NSAID* non-steroidal anti-inflammatory drugs

In the training set based on the Beijing cohort, 2014 (incidence, 6.9%) hospitalized patients developed AKI, in which 1632 (incidence, 5.6%) patients were diagnosed as stage 1 AKI and 765 (incidence, 2.6%) patients were diagnosed as stage 2 and 3 AKI. Patients who developed both stage 1 and stage 2 and 3 AKI were counted in both AKI stage 1 incidences and AKI stage 2 and 3 incidences. Besides, the number of cases of AKI in the internal validation set and the external validation set were 471 (incidence, 6.5%) and 33 (incidence, 6.2%) cases, respectively.

### Comparison of participants who developed acute kidney injury and those who did not

Supplementary Table 4 shows the demographics and clinical characteristics of patients in the derivation cohorts with and without AKI. Patients with AKI tended to be younger and had lower baseline creatinine values. Heart failure, respiratory failure, and pulmonary hypertension were significantly more prevalent in patients who developed AKI. Angiotensin-converting enzyme inhibitor and non-steroidal anti-inflammatory drug exposure was inversely correlated with the development of AKI. Diuretic and angiotensin receptor blocker drugs were associated with increased AKI risk. Those who developed AKI had lower platelet count, albumin and a higher white blood cell count.

### Performance of the prediction model

**Fig. 3:**
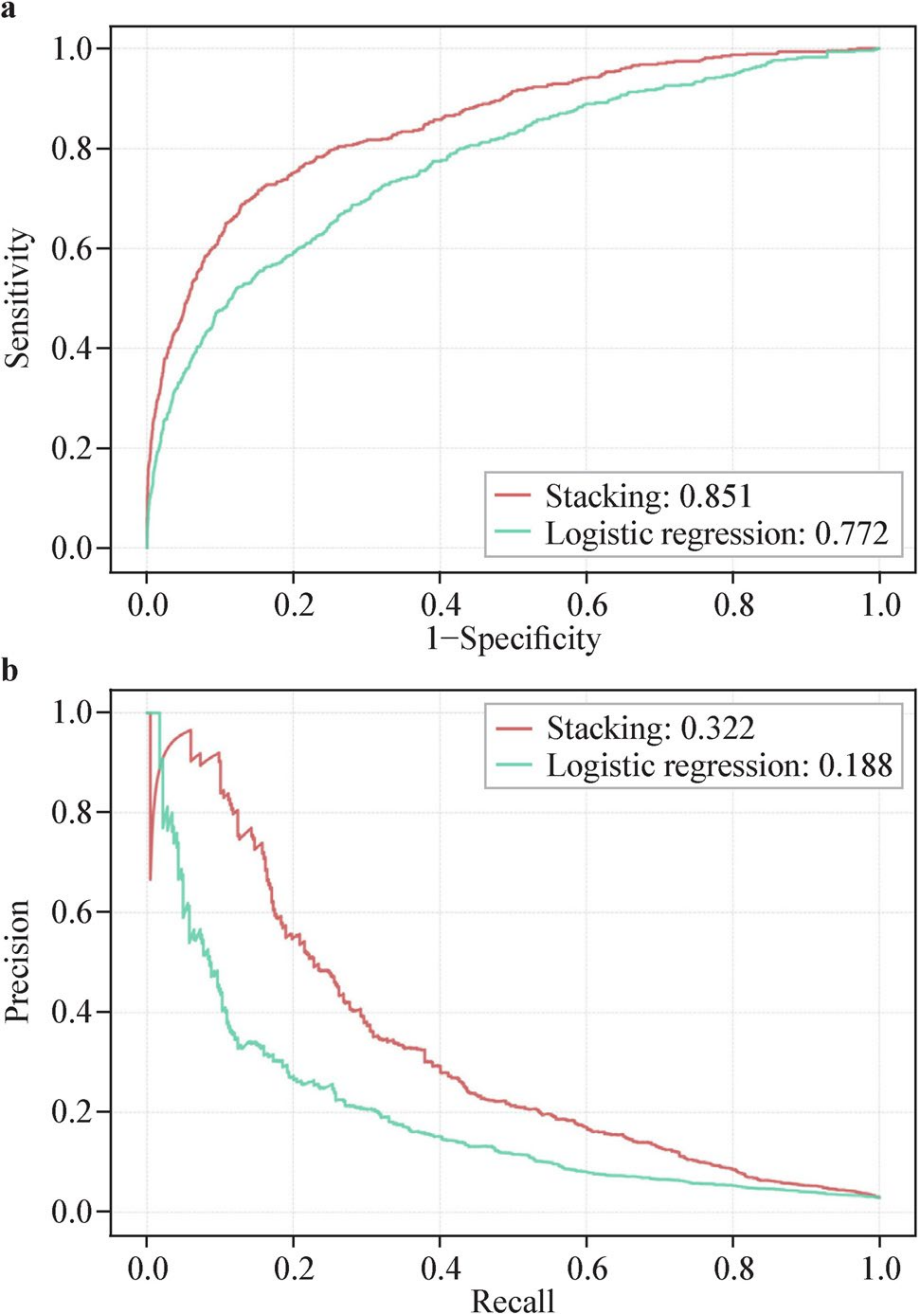
Predictive performance of different models on the internal validation set.** a** Receiver-operating curve. **b** Precision-recall curve

The receiver-operating curve for the prediction models which predicts the risk of any AKI development within 24 hours and the corresponding precision-recall curves are shown in Fig. 3. The stacking model (AUROC = 0.851, AUPR = 0.322) outperformed the logistic regression model (AUROC = 0.772, AUPR = 0.188). To obtain a stable estimation of performance indices, a 10 × tenfold cross-validation of the internal validation was also conducted to access the stacking model. As shown in Table 2, the mean of AUROC was 0.84 and the mean of AUPR was 0.31.

**Table 2:** The performance of prediction model estimated by cross validation

Prediction window	Precision	TP:FP	AUROC, mean (SD)	AUPR, mean (SD)	SEN, mean (SD)	SPE, mean (SD)	NPV, mean (SD)	F1-score, mean (SD)
Any AKI								
0–24 h	0.10	1:9	0.84 (0.013)	0.31 (0.030)	0.731 (0.0364)	0.790 (0.0020)	0.989 (0.0012)	0.176 (0.0011)
	0.20	1:4			0.495 (0.0511)	0.936 (0.0090)	0.983 (0.0015)	0.284 (0.0087)
	0.33	1:2			0.336 (0.0517)	0.979 (0.0039)	0.979 (0.0018)	0.332 (0.0267)
	0.50	1:1			0.217 (0.0453)	0.993 (0.0016)	0.976 (0.0020)	0.300 (0.0454)
24–48 h	0.10	1:9	0.82 (0.015)	0.29 (0.029)	0.703 (0.0476)	0.783 (0.0244)	0.987 (0.0017)	0.175 (0.0015)
	0.20	1:4			0.474 (0.0550)	0.935 (0.0097)	0.981 (0.0019)	0.280 (0.0098)
	0.33	1:2			0.308 (0.0477)	0.979 (0.0037)	0.976 (0.0020)	0.318 (0.0261)
	0.50	1:1			0.201 (0.0450)	0.993 (0.0017)	0.973 (0.0021)	0.284 (0.0457)
AKI stage 2 and 3								
0–24 h	0.10	1:9	0.87 (0.022)	0.35 (0.057)	0.618 (0.0708)	0.947 (0.0093)	0.996 (0.0007)	0.172 (0.0029)
	0.20	1:4			0.509 (0.0733)	0.981 (0.0037)	0.995 (0.0008)	0.286 (0.0126)
	0.33	1:2			0.421 (0.0741)	0.992 (0.0017)	0.994 (0.0008)	0.369 (0.0303)
	0.50	1:1			0.336 (0.0735)	0.997 (0.0008)	0.994 (0.0009)	0.397 (0.0559)
24–48 h	0.10	1:9	0.86 (0.024)	0.34 (0.060)	0.601 (0.0724)	0.943 (0.0108)	0.996 (0.0007)	0.171 (0.0031)
	0.20	1:4			0.486 (0.0717)	0.979 (0.0043)	0.995 (0.0008)	0.282 (0.0126)
	0.33	1:2			0.399 (0.0665)	0.992 (0.0019)	0.994 (0.0009)	0.360 (0.0286)
	0.50	1:1			0.316 (0.0714)	0.997 (0.0009)	0.993 (0.0010)	0.383 (0.0554)

*AKI* acute kidney injury, *TP* true positive, *FP* false positive, *AUROC* area under receiver-operating curve, *AUPR* area under precision-recall curve, *SEN* sensitivity, *SPE* specificity, *NPV* negative predictive value, *SD* standard deviation

In addition, the model exhibited a better performance in predicting AKI stage 2 and 3, with an AUROC of 0.871 and an AUPR of 0.349. Another important scenario is to predict AKI risk at least 24 hours in advance. When the prediction time window was extended to 24–48 hours the model still performed well despite the increased difficulty in forecasting (Supplementary Fig. 3).

**Fig. 4:**
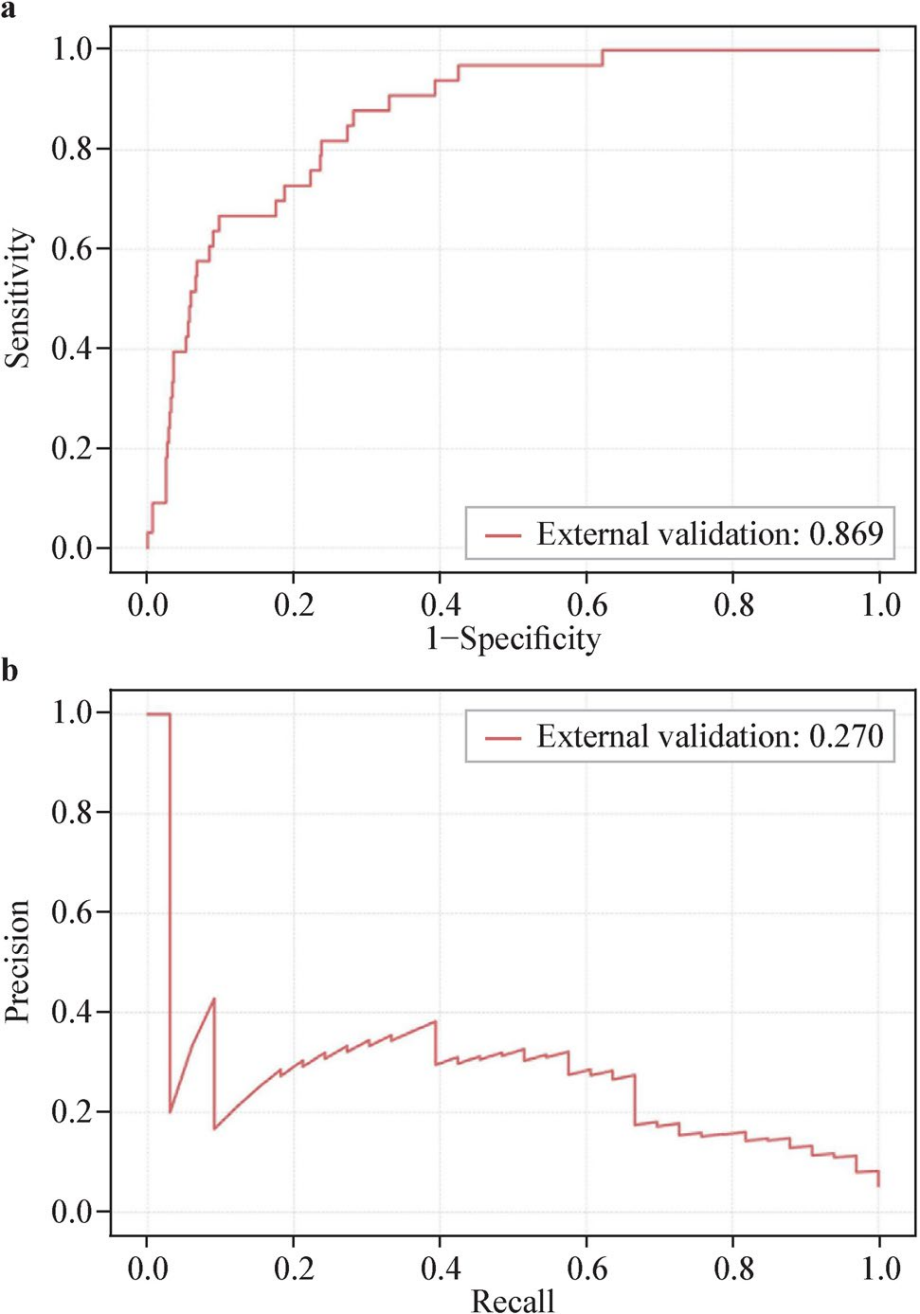
External validation of the stacking model. **a** Receiver-operating curve. **b** Precision-recall curve

Further external validation of the prediction model was performed based on the Chongqing cohort for any AKI in the prediction window of 0–24 hours. Similar indices were observed compared to the internal validation (Fig. 4) (AUROC = 0.869, AUPR = 0.270).

### Interpretation of variables

For interpretation of the model developed, SHAP values were used. The strongest predictors of AKI are listed in the SHAP summary bar plot (Supplementary Fig. 4a). The SHAP summary dot plot (Supplementary Fig. 4b) demonstrates the influence of each variable in terms of its impact on model predictions. On average, higher estimated glomerular filtration rate in baseline and higher creatinine and white blood cell values in the current time step are associated with a greater risk of AKI development. Surgery and heart failure are also important risk factors for predicting AKI.

In addition, we quantified the feature’s contribution to each individual prediction by analyzing local SHAP values (Supplementary Fig. 4c, d). Supplementary Fig. 4c shows the individual local SHAP values of a patient who developed AKI on the 7th day after admission. This patient had an increased phosphorus, platelet distribution width, heart rate, lactic dehydrogenase, and calcium; these results pushed the decision towards “AKI”. Supplementary Fig. 4d describes another patient who developed AKI on the 4th day after admission. The plot represents the patient moving towards the “AKI” with a probability of 0.748 on the 3rd day. Chloride, baseline estimated glomerular rate, sodium and phosphorus levels pushed the decision towards the “AKI”. Creatinine, current estimated glomerular rate values and weight pushed the decision towards “non-AKI”.

## Discussion

In the present study, a real-time prediction model of AKI for hospitalized pediatric patients was developed based on routinely collected health data. As recommended at the 15th Acute Dialysis Quality Initiative Consensus Conference, this prediction model of pediatric AKI could continuously and automatically monitor and assess the risk of pediatric patients developing AKI. As previously reported, several prediction models have been developed for the prediction of AKI. However, these studies specifically focused on AKI in a pre-specified fixed prediction window. A fixed prediction window makes it suitable only for a specific population who developed AKI within the first few days after a time point, e.g., the (pediatric intensive care unit) PICU population. For inpatients in general wards, the fixed prediction window model usually performed relatively poorly.

A strength of the present study is that the ensemble learning technique was used to address the class-imbalance issue of the data set. To improve performance of the model, a stacking technique was used to build an ensemble learning model, in which XGBoost, CatBoost and LightGBM classifiers were utilized as base learners. It is known that tuning the hyperparameters of each base learner inside the stacking model can lead to a better performance [26, 27, 39]. A large number of studies have shown that PSO has the properties of faster convergence and stronger global search capability compared to other optimization strategies [40]. Accordingly, the hyperparameters of the stacking model were optimized by the PSO algorithm. The proposed model based on the stacking technique showed relatively good performance.

An additional strength of the present study is that the developed prediction model of pediatric AKI also performs well in the external validation. Despite the variability in baseline characteristics—e.g., duration of hospital stay, spectrum of comorbidities at admission and drug exposure—between the external validation set from the Chongqing cohort and the training and internal validation sets from the Beijing cohort, the model demonstrates a robust performance with AUROC and AUPR values of 0.869 and 0.270, respectively. These metrics closely align with those observed in the internal validation, underscoring robustness of the model and potential applicability across diverse clinical settings.

It is widely acknowledged that the mechanisms underlying predictions made by machine learning models are complex and challenging to comprehend; this impedes integration into clinical practice [41–43]. Consequently, a further strength of this study is our endeavor to elucidate the clinical implications of prediction models. As shown in Supplementary Fig. 4, SHAP values revealed that the developed prediction model covered a range of well-established risk factors, all of which exhibited higher global average absolute SHAP values than other factors. Notably, local SHAP values also highlighted that AKI was presented as a syndrome with complex clinical presentation. It is essential to comprehensively evaluate risk factors and to consider their interactions, e.g., similar albumin values have different effects on AKI risk for the two patients in Supplementary Fig. 4c–d.

Furthermore, we extend the prediction window to 24–48 hours to leave a lead time for preventative actions to be taken. It is known that lead time improved the practicability and usefulness of machine learning model in clinical practice [44]. To this end, a lead time of 24 hours was considered, which means that the proposed model would generate AKI risk prediction at least 24 hours before occurrence.

There are some limitations associated with this study. First, the utilization of retrospective data for model training and validation necessitates further corroboration through a large-scale prospective study. Second, our model is updated daily, meaning that the AKI cases on the first day of admission could not be predicted. Nevertheless, only a small proportion of pediatric patients developed AKI on the first day in general wards. Third, only serum creatinine was used to define AKI due to data availability, potentially underestimating AKI rates compared to diagnoses that include urine criteria. Fourth, the proposed model did not account for varying types of surgeries, because extracting specific surgery types from the database was challenging. Including major surgeries, such as cardiac surgery could be beneficial. Fifth, defining a suitable cut-off point for the prediction model is a trade-off. The precision was limited to no less than 0.1 in our study to ensure that the number of false positive alarms was controlled. This has the disadvantage of reducing model sensitivity and results in some missing diagnosed pediatric patients.

In conclusion, this study introduced a machine learning approach for the real-time prediction of AKI risk for hospitalized pediatric patients. Validated with both internal and external datasets, this model successfully identified pediatric patients at risk of AKI, and demonstrated its potential applicability in clinical settings. The established risk predicting model can be used for real-time prevention of AKI in hospitalized pediatric patients.

## Supplementary Information

Below is the link to the electronic supplementary material.

Supplementary file1 (DOCX 1745 KB)

## Data Availability

The data are available from the corresponding author on reasonable request.
