# Supplementary material for: Development and validation of a real-time risk prediction model for acute kidney injury in hospitalized pediatric patients
Source: World J Pediatr. 2025 Jul 30;21(9):878–88. doi: 10.1007/s12519-025-00950-2 (PMC12433335; doi:10.1007/s12519-025-00950-2)
Supplement: Supplementary file 1 — Supplementary file1 (DOCX 1745 KB) [file 12519_2025_950_MOESM1_ESM.docx]

**Development and validation of a real-time risk prediction model for acute kidney injury in hospitalized pediatric patients**

Chao Zhang^1^, Chen Wang^1^, Qin-Shi Hu^2^, Xi-Ming Xu^2^, Ruo-Hua Yan^1^, Xiao-Lu Nie^1^, Ya-Guang Peng^1^, Hai-Ping Yang^3^, Yao Song^4^, Xue-Jun Yang^3^, Xiao-Xia Peng^1^

1 Department of Clinical Epidemiology and Evidence-based Medicine, Beijing Children’s Hospital, Capital Medical University, National Center for Children Health, Beijing, China

2 Big Data Center for Children’s Medical Care, Children’s Hospital of Chongqing Medical University, Chongqing, China

3 Department of Nephrology, Children’s Hospital of Chongqing Medical University, National Clinical Research Center for Child Health and Disorders, Ministry of Education Key Laboratory of Child Development and Disorders, Chongqing Key Laboratory of Pediatric Metabolism and Inflammatory Disease, Chongqing, China

4 Department of Biostatistics, University of Michigan, Ann Arbor, USA

**Supplementary Materials**

| Figure S1. Predictive performance of the simplified model. | 3 |
| --- | --- |
| Figure S2. Architectural overview of the developed model using the stacking technique and PSO algorithm. | 4 |
| Figure S3. Internal validation of the stacking model on different prediction windows and AKI stages. | 5 |
| Figure S4. Model explanation by the SHAP method. | 6 |
| Table S1. The list of variables. | 8 |
| Table S2. The list of variables for the simplified prediction model. | 9 |
| Table S3. Hyperparameters of the stacking model. | 10 |
| Table S4. Comparison of demographic and clinical characteristics between AKI group and non-AKI group in derivation set. | 11 |
| Table S5. Model performance for predicting AKI on subgroups estimated by cross validation. | 13 |


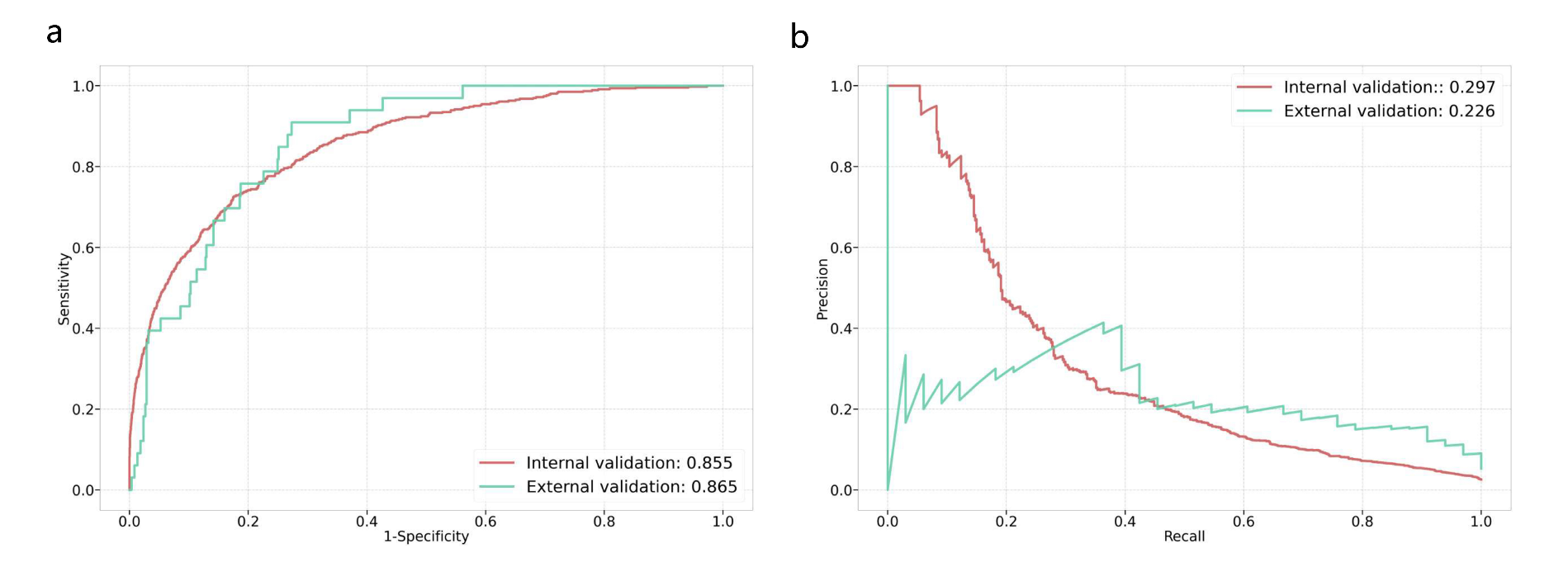


**Fig. S1: Predictive performance of the simplified model.**

(a) receiver-operating curve. (b) precision-recall curve.


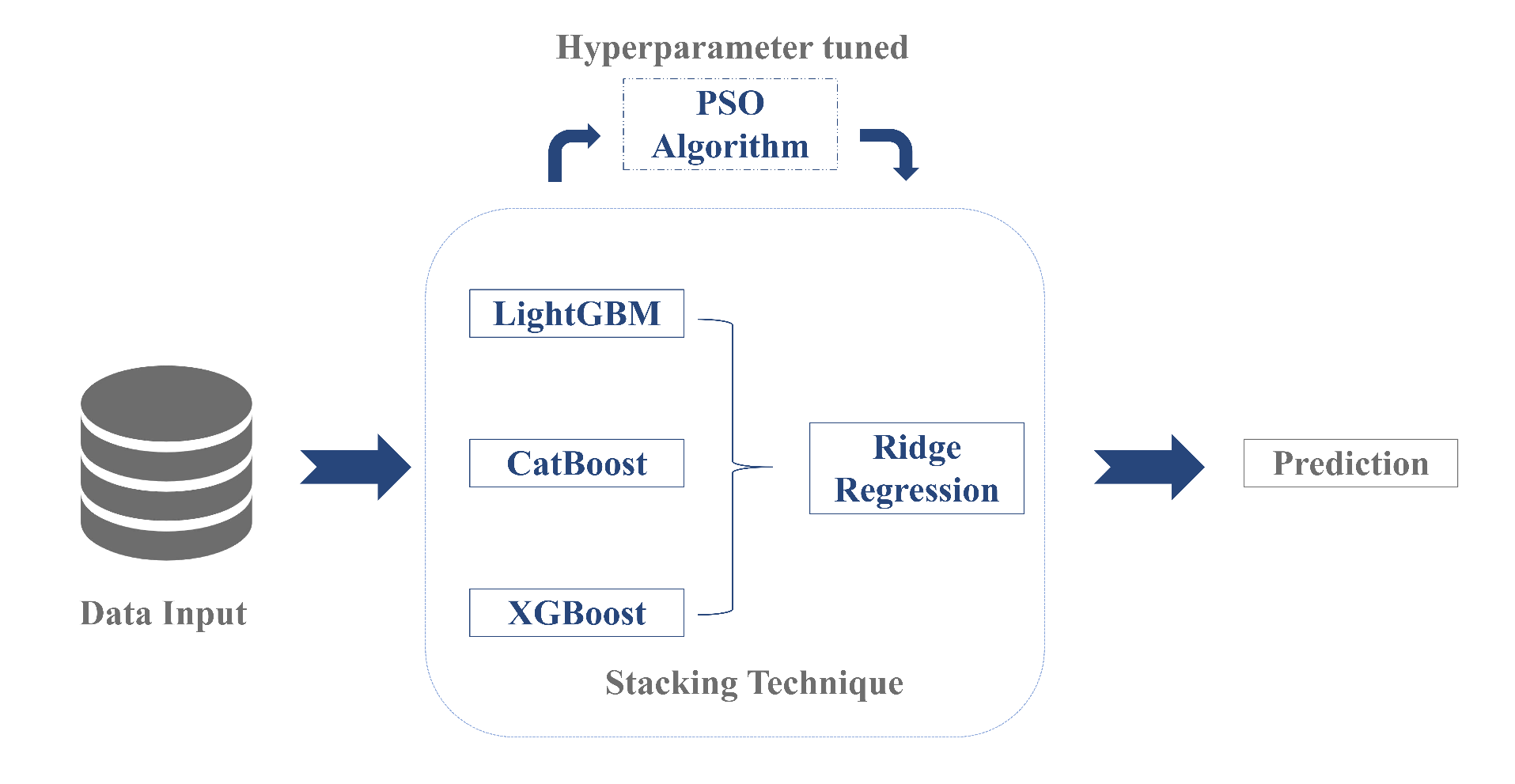


**Fig. S2: Architectural overview of the developed model using the stacking technique and PSO algorithm.**

Abbreviations: PSO: particle swarm optimization.


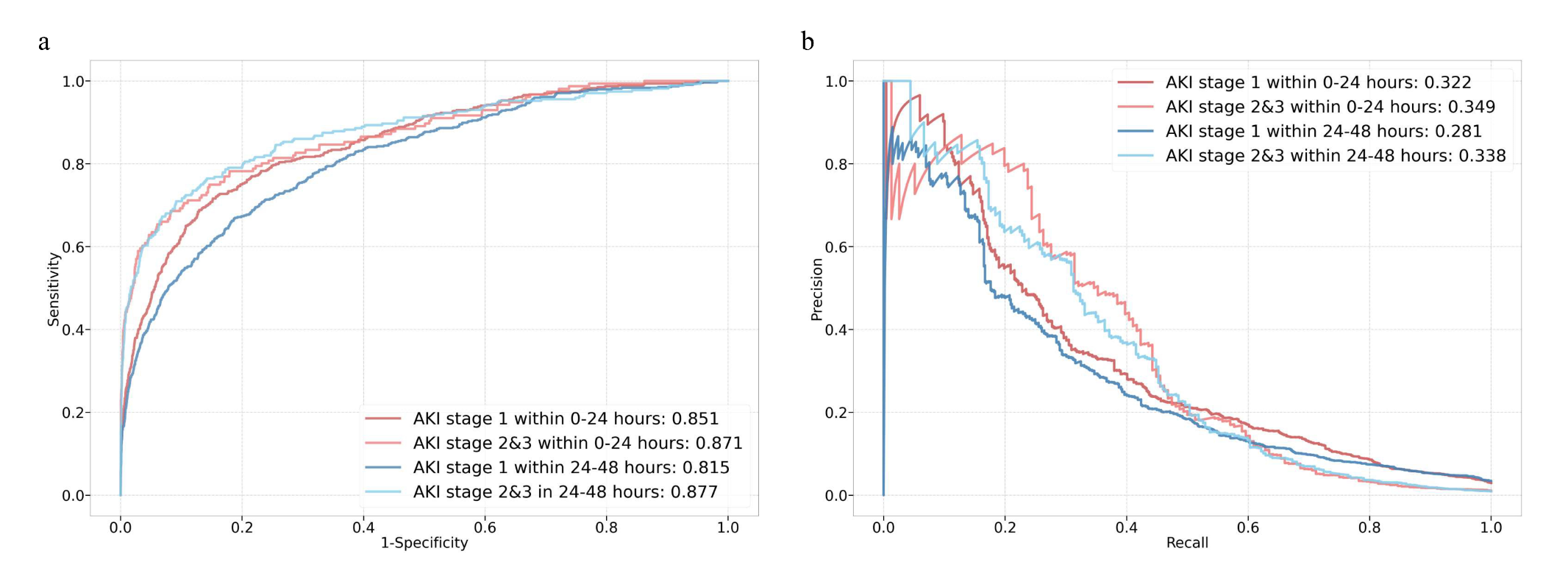


**Fig. S3:** **Internal validation of the stacking model on different prediction windows and AKI stages.**

(a) receiver-operating curve. (b) precision-recall curve.

Abbreviations: AKI: acute kidney injury.


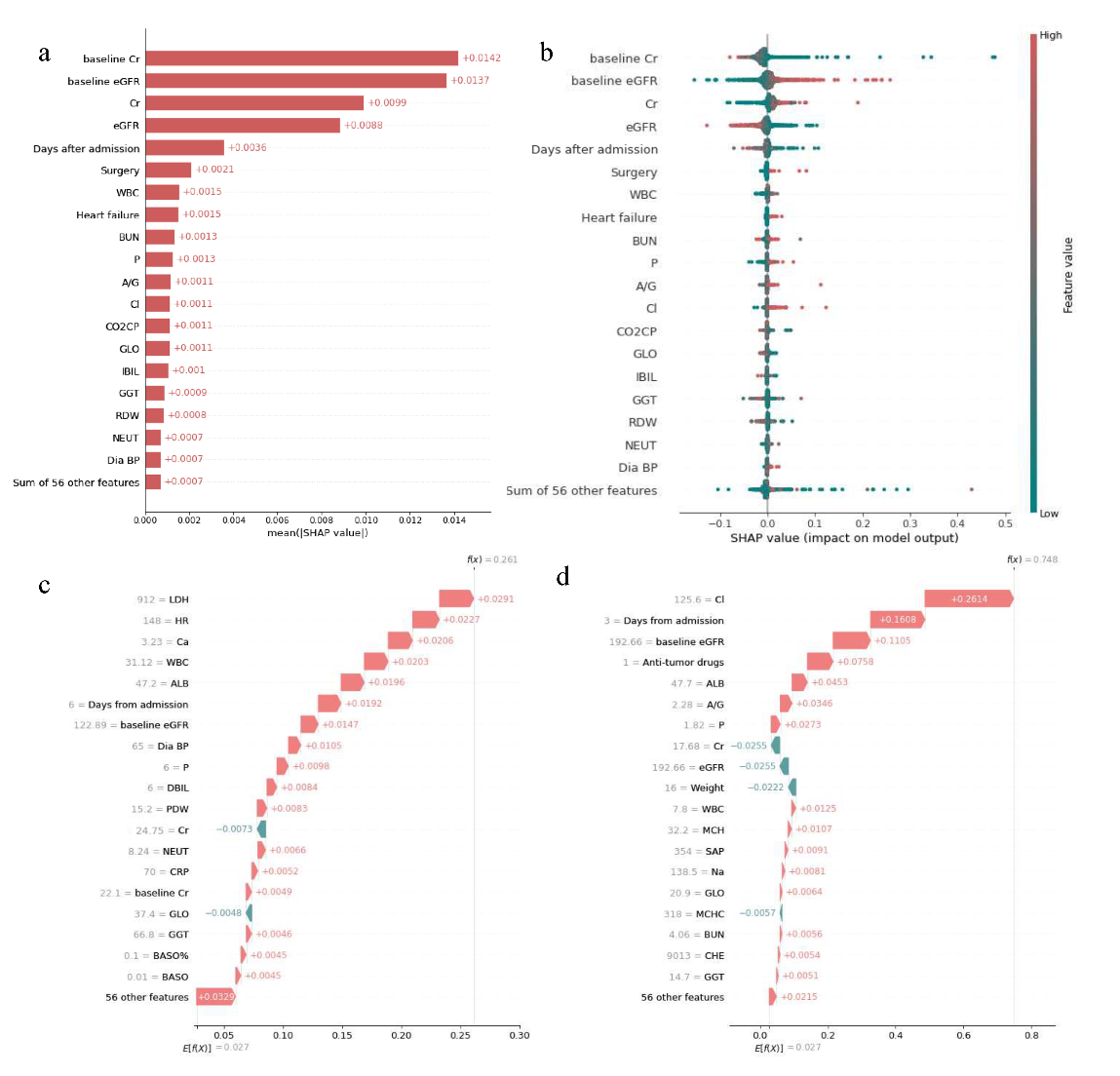


**Fig. S4: Model explanation by the SHAP method.**

(a) SHAP summary bar plot. The bar plot shows the importance of each feature based on the absolute mean value of the SHAP value. (b) SHAP summary dot plot. In the summary dot plot, each sample is represented as a single dot, and the position is determined by its SHAP value, while the color indicates its original feature value. Red signifies a higher feature value and blue signifies a lower feature value. (c) SHAP waterfall plot of a child who developed AKI. (d) SHAP waterfall plot of another child who developed AKI.

Abbreviations: A/G: albumin/globulin ratio; ALB: albumin; ALP: alkaline phosphatase ; BASO%: basophil percentage; BUN: blood urea nitrogen; Ca: calcium; CHE: cholinesterase; Cl: chloride; CO2CP: carbon dioxide combining power; Cr: creatinine; DBIL: direct bilirubin; Dia BP: diastolic blood pressure; eGFR: estimated glomerular filtration rate; EO: eosinophil count; EO: eosinophil percentage; GGT: gamma-glutamyl transferase; GLO: globulin content; HR: heart rate; IBIL: indirect bilirubin; LDH: [lactic dehydrogenase](https://www.baidu.com/s?wd=lactic%20dehydrogenase%E7%BF%BB%E8%AF%91&rsv_idx=2&tn=baiduhome_pg&usm=1&ie=utf-8&rsv_pq=c40cceff001ae08a&oq=%E4%B9%B3%E9%85%B8%E8%84%B1%E6%B0%A2%E9%85%B6%E8%8B%B1%E6%96%87&rsv_t=d793NzThIW9eB9vfs5LnWpu6xzGvj5V0vvGEiHH7rRZvGXk9lIPHlicD5m%2Ft%2BvSvfiuB&sa=re_fy_huisou); MCH: mean corpuscular hemoglobin; MCHC: mean corpuscular hemoglobin concentration; Na: sodium; NEUT: neutrophil count; P: phosphorus; PDW: platelet distribution width; RDW: red blood cell distribution width; TBIL: total bilirubin; UA: urea acid; WBC: white blood cell.

**Table S1. The list of variables**

| **Type** |  | **Features** |  |
| --- | --- | --- | --- |
| **Demographics (4)** |  | Age, Gender, Weight, Hight |  |
| **Laboratory values (52)** |  | Albumin/globulin ratio, Blood urea nitrogen, Baseline blood urea nitrogen, Magnesium, Gamma-glutamyl transferase, White blood cell, Red blood cell, Platelet, Neutrophil count, Neutrophil percentage, Chloride, Phosphorus, Mean corpuscular volume, Mean corpuscular hemoglobin, Carbon dioxide combining power, Red blood cell distribution width, Globulin content, Calcium, Mean corpuscular hemoglobin concentration, L[actic dehydrogenase](https://www.baidu.com/s?wd=lactic%20dehydrogenase%E7%BF%BB%E8%AF%91&rsv_idx=2&tn=baiduhome_pg&usm=1&ie=utf-8&rsv_pq=c40cceff001ae08a&oq=%E4%B9%B3%E9%85%B8%E8%84%B1%E6%B0%A2%E9%85%B6%E8%8B%B1%E6%96%87&rsv_t=d793NzThIW9eB9vfs5LnWpu6xzGvj5V0vvGEiHH7rRZvGXk9lIPHlicD5m%2Ft%2BvSvfiuB&sa=re_fy_huisou), Direct bilirubin, Total bilirubin, Indirect bilirubin, Hemoglobin, Glucose, Total protein, Albumin, Lymphocyte percentage, Lymphocyte count, Basophil count, Basophil percentage, Monocyte count, Monocyte percentage, Eosinophil count, Eosinophil percentage, Creatinine, Baseline creatinine, Estimated glomerular filtration rate, Baseline estimated glomerular filtration rate, Urea acid, Potassium, Aspartate transaminase, Alanine transaminase, Aspartate transaminase/ Alanine transaminase ratio, Sodium, Alkaline phosphatase, Cholinesterase, Prealbumin, Total bile acid, Platelet distribution width, Procalcitonin, C-reactive protein |  |
| **Vitals (4)** |  | Heart rate, Temperature, Systolic pressure, Diastolic pressure |  |
| **Medications (7)** |  | Aminoglycosides, Angiotensin converting enzyme inhibitor, Angiotensin receptor blocker  , Nonsteroidal anti-inflammatory drugs, Diuretics, Anti-tumor drugs, Acyclovir, |  |
| **Comorbidities (6)** |  | Heart failure, Respiratory failure, Septicemia, Pulmonary hypertension, Diabetes, Hypertension |  |
| **Other (2)** |  | Surgery, Days from admission |  |

**Table S2. The list of variables for the simplified model.**

| **Type** |  | **Features** |  |
| --- | --- | --- | --- |
| **Demographics (1)** |  | Weight |  |
| **Laboratory values (20)** |  | Albumin/globulin ratio, Gamma-glutamyl transferase, White blood cell, Red blood cell, Phosphorus, Potassium, Chloride, Calcium, Hemoglobin, Aspartate transaminase/ Alanine transaminase ratio, Urea acid, Albumin, Carbon dioxide combining power, L[actic dehydrogenase](https://www.baidu.com/s?wd=lactic%20dehydrogenase%E7%BF%BB%E8%AF%91&rsv_idx=2&tn=baiduhome_pg&usm=1&ie=utf-8&rsv_pq=c40cceff001ae08a&oq=%E4%B9%B3%E9%85%B8%E8%84%B1%E6%B0%A2%E9%85%B6%E8%8B%B1%E6%96%87&rsv_t=d793NzThIW9eB9vfs5LnWpu6xzGvj5V0vvGEiHH7rRZvGXk9lIPHlicD5m%2Ft%2BvSvfiuB&sa=re_fy_huisou), Glucose, Red blood cell distribution width, Creatinine, Baseline creatinine, Estimated glomerular filtration rate, Baseline estimated glomerular filtration rate |  |
| **Vitals (1)** |  | Heart rate |  |
| **Medications (1)** |  | Nonsteroidal anti-inflammatory drugs |  |
| **Comorbidities (2)** |  | Heart failure, Pulmonary hypertension |  |
| **Other (1)** |  | Surgery |  |

**Table S3. Hyperparameters of the stacking model.**

| **Base learners** | **Hyperparameters** | Values |
| --- | --- | --- |
| **CatBoost** | Number of iterations | 1000 |
|  | Learning rate | 0.022 |
|  | Max depth | 5 |
| **XGBoost** | Number of iterations | 850 |
|  | Learning rate | 0.0132 |
|  | Max depth | 8 |
| **LightGBM** | Number of iterations | 600 |
|  | Learning rate | 0.0132 |
|  | Max depth | 8 |

The PSO algorithm was conducted using “Scikit-opt” package.

**Table S4. Comparison of demographic and clinical characteristics between AKI group and non-AKI group in derivation set.**

|  | **Non-AKI (n=27 062)** | **AKI (n=2014)** | **P value** |
| --- | --- | --- | --- |
| **Gender M** | 15 597 (57.6) | 1183 (58.7) | 0.3331 |
| **Age** | 4.8 (1.9, 9.1) | 3.3 (0.9, 7.5) | <0.0001 |
| **Laboratory values** |  |  |  |
| **Baseline Cr (μmol/L), Median (IQR)** | 28 (21, 37) | 21 (15, 29) | <0.0001 |
| **Baseline eGFR (15 mL/min/1.73m2), Median (IQR)** | 114 (86, 140) | 122 (74, 177) | <0.0001 |
| **Cr (μmol/L), Median (IQR)** | 27 (21, 36) | 22 (16, 33) | <0.0001 |
| **eGFR (15 mL/min/1.73m2), Median (IQR)** | 123 (99, 148) | 127 (83, 178) | 0.0190 |
| **WBC (10^9/L), Median (IQR)** | 6.9 (4.2, 10.3) | 8.0 (4.5, 12.2) | <0.0001 |
| **P (mmol/L), Median (IQR)** | 1.5 (1.3, 1.7) | 1.5 (1.2, 1.8) | 0.0444 |
| **PLT (10^9/L), Median (IQR)** | 289 (171, 406) | 244 (119, 381) | <0.0001 |
| **ALB (g/L), Median (IQR)** | 37 (33, 41) | 36 (31, 41) | <0.0001 |
| **Comorbidities, N (%)** |  |  |  |
| **Diabetes** | 1316 (4.9) | 98 (4.9) | 0.9951 |
| **Heart failure** | 964 (3.6) | 181 (9.0) | <0.0001 |
| **Hypertension** | 1801 (6.7) | 158 (7.9) | 0.0398 |
| **Pulmonary hypertension** | 387 (1.4) | 110 (5.5) | <0.0001 |
| **Respiratory failure** | 1626 (6.0) | 278 (13.8) | <0.0001 |
| **Septicemia** | 1122 (4.2) | 94 (4.7) | 0.2595 |
| **Drugs, N (%)** |  |  |  |
| **ACEI** | 1507 (5.6) | 86 (4.3) | 0.0135 |
| **Acyclovir** | 1501 (5.6) | 122 (6.1) | 0.3351 |
| **AGs** | 522 (1.9) | 31 (1.5) | 0.2168 |
| **Anti-tumor** | 5930 (21.9) | 462 (22.9) | 0.2831 |
| **ARB** | 43 (0.2) | 12 (0.6) | <0.0001 |
| **Diuretics** | 1040 (3.8) | 122 (6.1) | <0.0001 |
| **NSAID** | 8945 (33.1) | 617 (30.6) | 0.0258 |

Note: The laboratory values were the most recent results before AKI occurred for AKI group or the most recent results before discharging for non-AKI group. The differences between groups of categorical variables were compared using the Chi-square test or Fisher’s exact test, and that of continuous variables were compared with T-test or Wilcoxon rank sum test.

Abbreviations: ACEI: angiotensin-converting enzyme inhibitor; AGs: amino glycosides; ALB: albumin; ARB: angiotensin receptor blocker; Cr: creatinine; eGFR: estimated glomerular filtration rate; NSAID: non-steroidal anti-inflammatory drugs; P: phosphorus; PLT: Platelet; WBC: white blood cell.

**Table S5. Model performance for predicting AKI on subgroups estimated by cross validation.**

| **Any AKI** | | | | | |
| --- | --- | --- | --- | --- | --- |
| **Subgroup** | **AUROC, Mean (SD)** | **AUPR, Mean (SD)** | **SEN, Mean (SD)** | **SPE, Mean (SD)** | **NPV, Mean (SD)** |
| Age<=2years | 0.84 (0.014) | 0.35 (0.038) | 0.587 (0.0429) | 0.899 (0.0140) | 0.981 (0.0017) |
| Age>2years | 0.83 (0.011) | 0.26 (0.021) | 0.410 (0.0473) | 0.958 (0.0043) | 0.984 (0.0019) |
| Male | 0.84 (0.011) | 0.31 (0.022) | 0.487 (0.0410) | 0.938 (0.0066) | 0.983 (0.0012) |
| Female | 0.83 (0.014) | 0.30 (0.032) | 0.502 (0.0463) | 0.936 (0.0079) | 0.983 (0.0015) |
| **AKI stage2 & 3** | | | | | |
| **Subgroup** | **AUROC, Mean (SD)** | **AUPR, Mean (SD)** | **SEN, Mean (SD)** | **SPE, Mean (SD)** | **NPV, Mean (SD)** |
| Age<=2years | 0.86 (0.024) | 0.39 (0.057) | 0.526 (0.0628) | 0.971 (0.0065) | 0.993 (0.0009) |
| Age>2years | 0.87 (0.018) | 0.31 (0.044) | 0.481 (0.0505) | 0.986 (0.0024) | 0.996 (0.0004) |
| Male | 0.87 (0.018) | 0.36 (0.049) | 0.503 (0.0432) | 0.980 (0.0023) | 0.995 (0.0005) |
| Female | 0.86 (0.025) | 0.33 (0.079) | 0.498 (0.0864) | 0.982 (0.0044) | 0.995 (0.0009) |

Note: Subgroup analysis was conducted with a precision of 0.2.

Abbreviations: AKI: acute kidney injury; AUROC: area under receiver-operating curve; AUPR: area under precision-recall curve; SEN: sensitivity; SPE: specificity; NPV: negative predictive value; SD: standard deviation.
